# Life after Lockdown: An Exploratory Qualitative Study of Behaviors and Impacts of Avoiding COVID-19 in Individuals at High Risk of Severe COVID-19 and Their Caregivers

**DOI:** 10.3390/ijerph21101307

**Published:** 2024-09-30

**Authors:** Tiago Maia, Renata Yokota, Sofie Arnetorp, Joanne Smith, Gail Rae-Garwood, Gabriella Settergren, Marie Eckerd, Paul Williams

**Affiliations:** 1 Patient Centered Solutions, IQVIA, 2740-266 Porto Salvo, Portugal; tiago.maia@iqvia.com; 2 Epidemiology & Pharmacovigilance, P95, 3001 Leuven, Belgium; renata.yokota@astrazeneca.com; 3 Health Economics & Payer Evidence, Vaccines & Immune Therapies, BioPharmaceuticals Medical, AstraZeneca, 431 83 Gothenburg, Sweden; sofie.arnetorp@astrazeneca.com; 4 Patient representative, Vale of Glamorgan, UK; jo.smith73@hotmail.com; 5 Patient representative, Glendale, AZ, USA; slowitdownckd@gmail.com; 6 Global Evidence Portfolio Delivery, BioPharmaceuticals Medical, AstraZeneca, 431 83 Gothenburg, Sweden; gabriella.settergren@astrazeneca.com; 7 Global Patient Engagement R&I, AstraZeneca, Wilmington, DE 19850, USA; marieeckerd4@gmail.com; 8 Patient Centered Solutions, IQVIA, 92400 Courbevoie, France; 9 Patient Centered Science, Vaccine & Immune Therapies, BioPharmaceuticals Medical, AstraZeneca, 431 83 Gothenburg, Sweden

**Keywords:** physical distancing, quality of life, conceptual model, immunocompromised, focus group, SARS-CoV-2

## Abstract

This exploratory qualitative study involved semi-structured interviews with adults and caregivers of adults at high risk of severe COVID-19, addressing current COVID-19 avoidance and protective behaviors and how these behaviors impacted their lives. Results were interpreted in a separate think tank session. Insights were developed into a conceptual model of COVID-19 avoidance and protective behaviors and the associated impacts on health-related quality of life and overall functioning. Data were interpreted using a hybrid inductive and deductive thematic analysis. Twelve high-risk individuals and two caregivers were interviewed across four focus groups (April–July 2022). Major behavioral themes included physical distancing, physical and medical protection, quality of support services and networks, and information to make decisions. Major impacts included family, social, and emotional functioning; work and finances; and healthcare access. The final conceptual model comprised 13 behaviors to avoid COVID-19 categorized within four themes, and 13 impacts within five themes. Individuals at high risk of severe COVID-19 and their caregivers continue practicing COVID-19 avoidance behaviors post-lockdown and feel left behind by the general population. Our conceptual model may be used to inform health authorities and other governing bodies’ decisions in executing strategies aimed at improving these individuals’ lives.

## 1. Introduction

Early in the COVID-19 pandemic, certain populations (for example, immunocompromised individuals, the elderly, or those with certain health conditions) were identified as being at high risk of severe COVID-19 and advised to practice stringent self-protective behaviors, including self-isolation and physical distancing [1,2]. However, studies conducted during periods of government-imposed COVID-19-related restrictions (referred to as ‘lockdowns’ hereafter) demonstrated variability in the continuity of care for these individuals, as well as increased reliance on telemedicine [3]. This reportedly eroded high-risk individuals’ and clinicians’ confidence in the delivery and quality of care patients received [3]. Moreover, reductions in health-related quality of life (HRQoL) were observed in high-risk individuals, due in large part to elevated levels of stress, anxiety, loneliness, and depression, as well as a reduction in sleep quality and overall physical activity [3,4,5,6,7,8,9]. Similarly, caregivers (both professional and informal/family) of high-risk individuals experienced negative impacts on their own physical and psychological health, such as stress, anxiety, loneliness, and depression [10].

Due in large part to the implementation of vaccination programs, which proved effective for the general population, lockdown restrictions in the UK were lifted by February 2022 [11], and in May 2023, the World Health Organization declared an end to the global health emergency, and the US Centers for Disease Control and Prevention (US CDC) announced the end of the federal COVID-19 public health emergency in the United States [12,13]. While daily life returned to normal for the general population, COVID-19 remains a threat to people who are at high risk for severe COVID-19, especially immunocompromised individuals, owing to their suboptimal response to vaccines.

Data collected in 2022 from the UK Office for National Statistics showed that 69% of people at high risk for severe COVID-19 still took extra precautions, and 13% continued to follow previous government advice [14]. A national survey conducted in the Netherlands in 2023 revealed that one in four immunocompromised individuals were still ‘shielding’ (shielding is a term used in the UK and Europe for the specific act of employing multiple avoidance and protective strategies following government-recommended guidance; the act of shielding is to protect oneself, under the context of having no available pharmacological protection, e.g., vaccination) [15]. Furthermore, the US CDC and UK Department of Health and Social Care still advise high-risk individuals to stay up to date on vaccines, and continue to take preventative measures, such as masking in public and around friends/family and practicing physical distancing [16,17]. 

While physical distancing measures have proven effective in reducing exposure to SARS-CoV-2 in high-risk populations, the negative impacts of physical distancing may still be experienced by these individuals, although there are limited data exploring their extent. In a national UK survey conducted in July 2023, immunocompromised individuals facing a fourth year of practicing physical distancing reported reduced HRQoL compared with the general population [6]. Additionally, there is evidence that well-being among immunocompromised individuals has not returned to pre-COVID-19 levels, based on the national survey conducted in the Netherlands [15]. 

With the reallocation of government funds and attention away from COVID-19 pandemic relief and toward long-term economic recovery [18], studies have shown that high-risk populations feel ‘left behind’ and have lost trust in institutions, and some are dissatisfied with the current situation [6]. For example, during lockdown, hospitals put protective measures in place to prevent infection; post-lockdown, these are no longer in place, meaning that hospital visits are associated with higher risk [19,20,21,22]. It is important to understand the health effects, perspective, and experience of high-risk individuals continuing voluntary physical distancing post-lockdown, especially while most of the population no longer engages in restrictive behaviors. Understanding and acknowledging caregivers’ experience is also critical to providing equitable support, as they share the burden of continued physical-distancing measures and other care activities [10]. Raising awareness of these needs is necessary in order to provide policy/decision-makers and clinicians with useful and actionable insights to develop and implement plans that support both individuals at high risk of severe COVID-19 and their caregivers.

The aim of this study was to explore and qualitatively describe the COVID-19 avoidance and protective behaviors of individuals at high risk of severe COVID-19 and their caregivers, and the perceived HRQoL impact of these behaviors. The description of these behaviors and impacts at a time point long after national lockdowns have ended is intended to inform the conceptualization of the burden of disease for future studies investigating the continued burden of COVID-19 in high-risk individuals.

## 2. Materials and Methods

### 2.1. Participant Recruitment

Participants were selected via a convenience sampling approach from a panel managed by AstraZeneca’s Patient Partnership Program. Guidelines recommend a sample size of two to four participants in a focus group [23,24]; therefore, a sample size of 14 participants across four focus groups was considered reasonable to ensure diversity among participants in terms of age, sex, and underlying disease.

Participants were contacted by email with an invitation to participate and were subsequently asked to provide their availability for the interview. Eligible participants were English-speaking adults and caregivers of adults (age ≥ 18 years) at high risk of severe COVID-19. High-risk individuals included those diagnosed with a medical condition that reduces immunity (e.g., chronic kidney disease, chronic obstructive pulmonary disorder, hepatocellular carcinoma, lupus, or kidney transplant). Ineligible participants included those with a significant disability, limited legal capacity, or any other lack of fitness that would hinder the participant’s ability to participate in the study.

### 2.2. Study Design

The qualitative focus groups were conducted as online interviews with individuals at high risk of severe COVID-19 and their caregivers. Focus groups were chosen over one-to-one interviews to promote the idea sharing and debate that naturally arise from spontaneous conversation, thereby enabling faster collection of information [25]. 

To investigate the evidence on patient-reported experiences of social isolation, physical distancing, and other protective behaviors to avoid COVID-19, as well as the association of these behaviors with HRQoL impacts, and to identify any patient-reported outcome measures used to measure these behaviors and impacts, we conducted a narrative review employing search strings designed for use in PubMed to capture published articles and studies from 1 December 2019 to 28 April 2022. The search terms used were as follows: COVID-19, shielding, shield, protection, isolation, impact, patient-reported outcomes, PRO, patient-reported, patient perspective, patient-centricity, patient-centered, interview, conceptual model, focus group, and qualitative. We searched for qualitative, quantitative, and mixed-methods studies, and the searches were limited to the English language. We found 373 references, of which 63 were included. Data on relevant HRQoL impacts reported due to COVID-19 avoidance behaviors, social isolation, shielding, and the overall impacts of COVID-19 on society were identified and listed. These findings were used to inform the study design, to identify a preliminary list of domains and specific impacts of social isolation to avoid COVID-19 (Table A1) and the overall impacts of the COVID-19 pandemic on society (Table A2), and to inform the questions asked during the four focus groups.

The primary objective of the focus groups was to explore and qualitatively describe the avoidance and protective behaviors of adults at high risk of severe COVID-19 and their caregivers, the factors influencing the choice to practice these behaviors, and the impact of these behaviors on their lives. The secondary objective was to develop a conceptual model of salient avoidance and protective behaviors to avoid COVID-19 and the associated impacts on HRQoL and overall functioning of these individuals. Behaviors were considered salient if mentioned by ≥30% of participants or if mentioned in all four focus group sessions. An additional exploratory objective was to describe the overarching impacts of the COVID-19 pandemic on society according to the experiences of the individuals at high risk of severe COVID-19 and their caregivers.

### 2.3. Data Collection

The four focus groups were conducted from April to July 2022 via videoconference by three trained researchers (TM, SA, and PW) following a predefined, semi-structured interview guide (see Appendix A Materials), and lasted approximately 60 min each. To ensure all participants could contribute equally and not be heavily influenced by group consensus (or ‘bandwagon’ effects), there were no more than four participants in each focus group (Focus Group [FG] 1: four participants; FG2: four participants; FG3: four participants; FG4: two participants). The groupings were determined by participant availability. During the focus group sessions, individuals at high risk of severe COVID-19 and their caregivers were asked a list of open-ended questions on topics related to their avoidance and protective behaviors and the impacts of those behaviors on their HRQoL in the post-lockdown period. Post-lockdown was defined as the period of time when COVID-19 was considered a pandemic, but after lockdown restrictions had been lifted in the UK, Spain, and Canada [11,26,27], and masking mandates had been lifted in the US, covering the focus group dates (April–July 2022; the final US travel restrictions were lifted in June 2022 [28,29]). The participants also reviewed lists of behaviors and impacts identified from other studies and commented on whether these applied to them. Verbatim transcripts of the focus groups were generated by two of the researchers, and all identifying information was removed. The third researcher reviewed the transcripts for accuracy and consistent reporting of the data captured. 

### 2.4. Qualitative Analysis

Data were interpreted using a hybrid inductive and deductive thematic analysis [30]. De-identified transcripts were analyzed by two independent researchers (one coder and one reviewer) who tagged each behavior or impact of avoiding COVID-19 with a code. A deductive approach (i.e., a process used to structure the coding and analysis based on data gathered from previous research [31]) to coding impacts on HRQoL due to COVID-19 avoidance was employed, with the development of a codebook informed by the literature review findings. An inductive approach (i.e., a process that includes open coding, grouping, and abstraction of new concepts not previously explored in earlier research [31]) was used to explore the avoidance and protective behaviors naturally emerging during coding. Coding was performed and qualitatively analyzed using MAXQDA 2022 (VERBI Software, 2021; VERBI Software GmbH, Berlin, Germany) to process the data. Quality checks were performed in MAXQDA 2022 to ensure the correct themes and concepts were captured and the appropriate nomenclature was applied. Data were then exported to Microsoft Excel for ease of data reporting and to support the generation of tables, figures, and lists.

### 2.5. Statistical Analysis

No formal statistical hypothesis testing or sample size calculation was conducted; all data reported are qualitative and descriptive; and categorical variables were reported as counts (frequencies and percentages) [23]. The sample size of 14 (across four separate focus group sessions) was considered reasonable to enable the identification of salient concepts from this heterogenous sample, based on the recommended sizes of two to four people per focus group [23,24]. This approach aimed to create a preliminary overall conceptual model; as such, there was no intention to reach saturation of concepts, nor was there an intention to make any inference relating to subgroups.

### 2.6. Think Tank Session

Findings from the focus groups were assessed in a think tank session by two individuals at high risk of severe COVID-19 and one caregiver. These individuals were participants in the AstraZeneca Patient Partnership Program (two of whom, JS and GRG, are patient-representative authors of this manuscript). A family member was present to support one of the research partners but was not involved in the assessment. The aim of this think tank session was to confirm that descriptions of salient avoidance and protective behaviors, and impacts on the participant’s HRQoL, were an accurate representation of the themes. Think tank research partners were asked if they continue to practice these behaviors, if there were any changes from the beginning of the COVID-19 pandemic compared with the present moment, the behaviors the research partners continue to practice the most, potential missing behaviors, the most impactful behaviors, and what their thoughts were about how this information should be used to create strategies to improve their lives in the future. The think tank was envisaged to promote people-centeredness and public and patient involvement in healthcare and research, through engagement with a group of suitable individuals capable of providing relevant insights for the discussion of findings according to their experience.

One of these research partner authors is a pancreatic cancer survivor who was diagnosed with chronic kidney disease in 2008. They recounted their own experience and that of their partner, stating that “the will to survive this [COVID-19] was 10 times stronger than surviving cancer. I have survived cancer and will not let this [COVID-19] claim me”. To protect themselves and each other, the author and their partner (who is immunocompromised) diligently practiced all recommended measures to avoid crowds by giving up date weekends, vacations, events/gatherings with friends and family, and many other activities.

Another research partner author is a caregiver for their children who each have complicated medical conditions. They expressed feelings that they have been ignored by the healthcare and schooling system, citing loss of family health records and failure to receive ‘shielding’ letters; delayed prescription/delivery of essential medication due to not being eligible for the home delivery program; poor access to healthcare; lack of consideration of their isolation/masking needs post-lockdown; and ambiguous and inconsistent advice from the government.

To avoid the potential for bias, the research partners in the think tank session were not involved in the four focus groups. During the think tank session, a conceptual model of behaviors to avoid COVID-19 and the associated impacts on HRQoL was developed based on the insights from the focus groups.

## 3. Results

### 3.1. Participant Demographics

Participant-reported demographics are summarized in Table 1. Fourteen participants who resided in the US, UK, Canada, and Spain were interviewed across four separate focus group sessions from April to July 2022. Twelve were at high risk of severe COVID-19, and two were family caregivers (a spouse of an individual with chronic kidney disease and a parent of an individual with liver cancer who were not involved in this study). Eight participants were female (57%).

### 3.2. Major Themes of COVID-19 Avoidance and Protective Behavior

**Figure 1:**
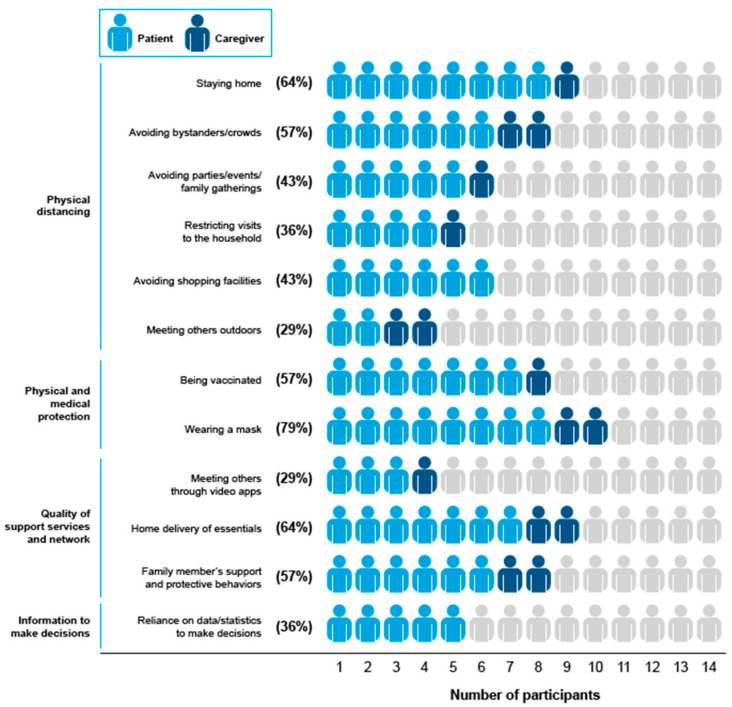
Salient physical distancing and protective behaviors mentioned by participants. Behaviors were considered salient if mentioned by ≥30% of participants or if mentioned in all four focus group sessions. Percentages are behaviors mentioned by patients or caregivers divided by the total number of participants (*N* = 14). A total of 33 behaviors were mentioned.

An overview of the 33 distinct avoidance and protective behaviors reported by all participants and caregivers is presented in Table A3 and Table A4. Of the 33 types of avoidance and protective behaviors reported, 12 were considered salient, 10 of which were due to being mentioned by ≥30% of participants, and two of which were due to being reported across all four focus groups. The most common behaviors reported by the 14 participants were wearing a mask (*n* = 11; 79%); home delivery of essentials and staying at home (each *n* = 9; 64%); avoiding bystanders and crowds, family members’ support and protective behaviors, and being vaccinated (each *n* = 8; 57%); avoiding parties, events, or family gatherings (*n* = 6; 43%); avoiding shopping facilities (*n* = 6; 43%); and reliance on data and statistics to make decisions on daily routine and restricting visits to the household (each *n* = 5; 36%). The two additional behaviors reported in all four focus groups included meeting others through video apps and meeting others outdoors. The frequencies of salient behaviors to avoid COVID-19 are presented in Figure 1, of which four major themes were identified: (1) physical distancing; (2) physical and medical protection; (3) quality of support services and networks; and (4) information to make decisions.

#### 3.2.1. Theme 1: Physical Distancing 

Participants reported that physical distancing took the form of behaviors to avoid or limit close proximity to people or specific situations or places where people may be present. These behaviors are listed in Figure 1.

Some participants mentioned seeing others exhibiting behavior that could potentially cause them harm; they did not feel comfortable being around others (including family) and expressed concern over their potential behaviors. Consequently, some participants felt that they had to limit their activities; avoid bystanders/crowds, going to large events (e.g., sports games and concerts), large family gatherings (e.g., weddings), and shopping facilities; and restrict visits to the household due to their perceived risk of being exposed to COVID-19. Several participants attended outdoor events to avoid putting themselves at risk.

*I honestly cannot remember an instance since the start of COVID when the last time was where we went to a baseball game…you don’t know who is going to brush by you or bump into you or sneeze on you.*

(Focus Group [FG]4 Patient [P]1)

*I grew very weary of people…I dodged the other way, crossed roads, stepped off the sidewalk…constantly turning around and walking the other way just not to get close to people.*

(FG2 P4)

*I think that has been the negative aspect of shielding…maybe too much time on my own…I’m not comfortable going into anybody else’s house or I definitely don’t like people coming to mine…I just think of all the things they would be breathing out.*

(FG2 P4)

#### 3.2.2. Theme 2: Physical and Medical Protection

Some participants (including one caregiver) reported that being vaccinated and wearing a mask were essential for them to feel comfortable in crowded places, shopping facilities, or when attending medical appointments.

*I wear masks in any kind of situation where there are more crowded people.*

(FG4 P2)

*When we attend medical appointments or hospital visits, that type of thing, masking is essential.*

(FG4 P1; caregiver of FG4 P2)

*I’ve now had four vaccinations, but I still am very, very cautious…it’s definitely a big part of my life about how I shield from COVID.*

(FG1 P3)

#### 3.2.3. Theme 3: Quality of Support Services and Networks

Several participants reported reliance on support services to allow them to remain in or adapt to the degree of isolation and avoid putting themselves at risk, such as attending or organizing online events, meeting others through video apps, using online messaging, and home delivery of essentials (e.g., food, necessities, and medication). At the same time, some participants felt the need to continue physically distancing themselves, despite being vaccinated, and that having to order everything and being unable to visit shopping facilities physically was isolating and depressing.

*I was ordering everything online…wiping down my groceries before I put them away in the cabinets and the fridge…my entire way of life changed exponentially…that was really, really difficult from a professional standpoint and personally it was so isolating and depressing.*

(FG2 P3)

Receiving support from families and having their family members practice social isolation behaviors to protect them were highlighted as important behaviors involving available support for some participants.

*The family network was important because we each became each other’s support in that instance while we were isolating…I just wonder what the situation would have been if perhaps it was a one-person house or by himself…without that support network.*

(FG3 P4)

#### 3.2.4. Theme 4: Information to Make Decisions

Several participants reported reliance on data/statistics to make decisions and that they actively consulted available data related to case evolution and number of infections to adapt their isolation accordingly.

*Last week was the highest in my area of people with COVID. Never been as high as that before…I stay indoors a lot more when the numbers (of COVID-19 cases) are high.*

(FG2 P1)

*I watch the numbers constantly, and when they’re higher I restrict myself more.*

(FG1 P2)

### 3.3. Major Themes of the Impact of Practicing Avoidance and Other Protective Behaviors to Avoid COVID-19

**Figure 2:**
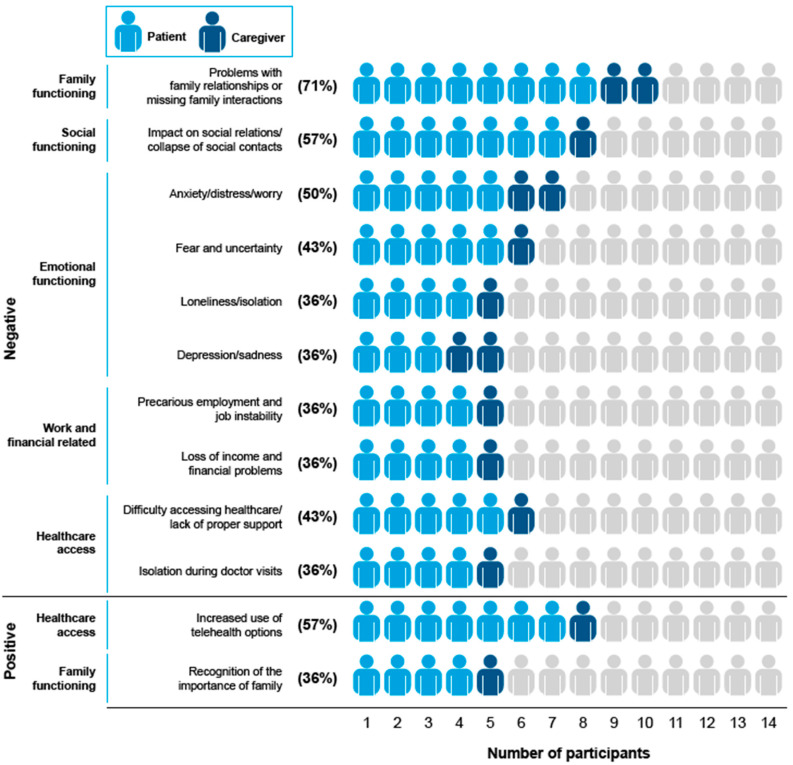
Salient positive and negative impacts of COVID-19 avoidance and protective behaviors reported by participants. Impacts were considered salient if mentioned by ≥30% of participants or if mentioned in all four focus group sessions. Percentages are impacts mentioned by patients or caregivers divided by the total number of participants (*N* = 14). A total of 40 negative impacts and 10 positive impacts were mentioned.

An overview of the 50 distinct impacts of COVID-19 avoidance and protective behaviors reported by all participants and caregivers is presented in Table A5 and Table A6. Of these, 40 were negative impacts of COVID-19 avoidance on overall HRQoL and overall functioning, and 10 were positive impacts on the participants’ lives. From the 50 distinct impacts elicited, 12 were considered salient (10 negative and 2 positive impacts), all of which were mentioned by ≥30% of participants. The frequencies of salient positive and negative impacts of COVID-19 avoidance behaviors reported by participants are presented in Figure 2. Five themes emerged upon evaluating the salient impact of participants’ behaviors: (1) family functioning; (2) social functioning; (3) emotional functioning; (4) work- and financial-related; and (5) healthcare access. The impact of physical functioning (walking problems due to isolation/lack of activity, inability to perform physical/outdoor hobbies, and lower levels of physical activity) was also discussed but not considered salient.

#### 3.3.1. Theme 1: Family Functioning

Salient negative impacts included problems with family relationships (e.g., family conflicts due to sharing the same space for long periods and having to reach agreement over how to isolate) and missing family interactions (e.g., not being able to meet family members and missing family gatherings/important family events [e.g., weddings and funerals] while isolating), while the salient positive impact was recognition of the importance of family.

*Things like family, missing family was awful. I’ve got a four-year-old grandson who doesn’t really know me…I’ve no relationship with him because we haven’t been able to meet up.*

(FG1 P2)

*Family conflicts…nobody could come into our house unless they were vaccinated…I have a brother-in-law that is an anti-vaxxer and refuses to wear a mask. He’s never been in our home.*

(FG1 P1)

#### 3.3.2. Theme 2: Social Functioning

The collapse of social relationships was a salient negative impact of social functioning, and no salient positive impacts of social functioning were reported.

*It was devastating to everyone when COVID hit and they made the decision do [sic] not return to (university) campus, we’re going to do it virtually, and it was just very difficult because when you leave home and go to, as a young person, a college that’s very small, and you’re living on campus and establishing friendships and relationships with other students and your professors, and all of a sudden that’s taken away from you. It was devastating.*

(FG1 P1)

#### 3.3.3. Theme 3: Emotional Functioning

Anxiety/distress/worry, fear and uncertainty, depression/sadness, and loneliness/isolation were the salient negative impacts of emotional functioning. Anxiety and worry were reportedly associated with participants’ lack of social contact, their fear of contacting others, and the risk that could entail (e.g., caregivers transmitting the disease to family members or friends). To cope with the isolation, participants went for walks or acquired specialist support for their mental well-being. No salient positive impacts of emotional functioning were reported.

*I was stressed all the time, every time I would go out…somebody would cough…. And you live with that fear…. The knowledge that if I got sick and gave it to him, he probably wouldn’t live through it.*

(FG1 P4)

*Social effects on me, [were] very negative…I felt myself slipping into depression…unable to do the things that I like to do…for two years, it’s had a huge impact.*

(FG1 P1)

#### 3.3.4. Theme 4: Work- and Financial-Related

Precarious employment and job instability, and loss of income and financial problems, were both reported as salient negative impacts related to work and finances. No positive salient work- or financial-related impacts were reported. Participants reported the need to stop working or shut down their business due to having to isolate, which led to financial struggles and disruptions to daily routines, social relationships, and emotional health. Financial problems were also linked by some participants to the overarching effects of the COVID-19 pandemic on markets and the economy.

*I was no longer able to bring in sufficient resources…relying on sick pay…was not sufficient income…that impacted on the finances of the household.*

(FG3 P4)

#### 3.3.5. Theme 5: Healthcare Access

Difficulty accessing/lack of healthcare support and isolation during doctor visits were salient negative impacts related to healthcare access, while the salient positive impact was the increased use of telehealth options. Participants reported that during lockdown, the negative impacts were hospital understaffing and the associated reduction in scheduled health appointments and restrictions on visitors during recovery. Fear of catching COVID-19 was also noted during lockdown; this persisted post-lockdown and continues to have an impact on the care and support the participants receive, particularly in terms of missing appointments.

*We always get regular notifications from our GP (general practitioner) to say ‘right, please don’t call us because we’re now short of staff and we’ve exceeded our hours that we’re actually safe to see patients’. We get texts probably twice a week to say we’ve withdrawn the online patient portal.*

(FG1 P3)

*If I got sick I didn’t want to go to the hospital with COVID because lot of doctors and nurses were working and treating COVID patients, and they were going to patient rooms that didn’t have COVID.*

(FG3 P2)

### 3.4. Exploratory Objective: Impact of the COVID-19 Pandemic on Society

Patients were additionally asked about perceived impacts to society; a total of two society-level impacts were identified as being salient: hoarding of products and medications (36%) and lack of trust in authorities/uncertainty over sources of information (57%). Participants reported difficulties in understanding the guidance from governments, their medical doctors, and scientific bodies (e.g., mask mandate rules), which led to a loss of confidence and a feeling of abandonment after lockdown restrictions were removed for the general population.

*In the early days we got the information from the government…. But…we’re on our own now…since all the restrictions were removed.*

(FG1 P2)

*The government were saying we’re following the science…and the government were ignoring it…there were times when you lost confidence in the government…the scientists…the medical professionals.*

(FG1 P3)

### 3.5. Patient/Caregiver Interpretations of the Results: Think Tank

During the think tank session, all research partners agreed that the avoidance and protective behaviors highlighted as salient (except for hoarding behaviors) were still being practiced in their own daily lives as high-risk individuals. Think tank research partners did highlight missing concepts, including hand washing and other hygiene measures as a means of physical and medical protection, and stigma or prejudice of others to the behaviors still practiced by participants to avoid COVID-19 as impacts on emotional functioning. The participants also prioritized avoidance and protective behaviors differently; one participant felt the key impact to consider was the difficulty in accessing healthcare and proper support, while another did not experience this problem due to the implementation of telehealth.

*My daughter’s hand washing actually got worse…she’s got OCD (obsessive compulsive disorder) now.*

(Think Tank Research Partner 3)

*I wear a mask, even outdoors, if there are crowds of people or groups of people, and we were in the park with the kids, I was wearing my mask and a kiddy birthday party came with lots of people and placed themselves right next to us…one woman was looking at me with horror on her face, and I didn’t know what was going on. Then when I got home, I realized I was the only one wearing the mask.*

(Think Tank Research Partner 1)

*There was one bloke who did have a go at me in the shop, actually made me feel very uncomfortable…. He wouldn’t move back for me to pass. “Excuse me”. And he was so rude to me, he said “go round the other way”…(saying) “If I have that concern (I) shouldn’t be in the shop” like, you know, but total ignorance.*

(Think Tank Research Partner 3)

### 3.6. Conceptual Model

**Figure 3:**
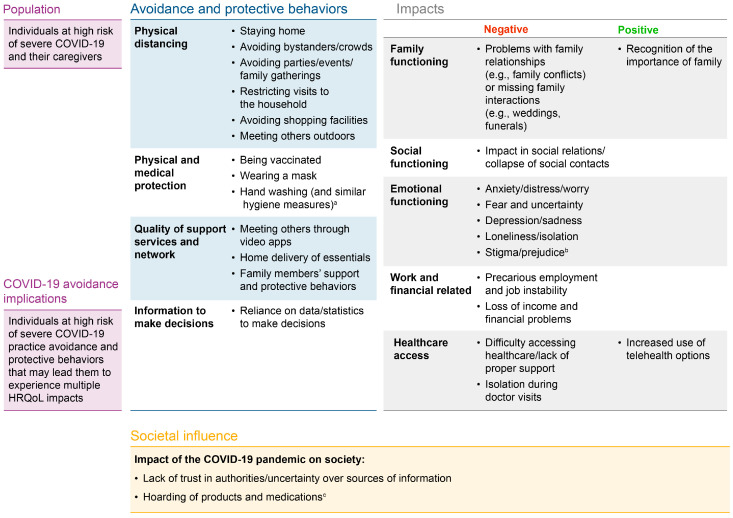
Conceptual model of avoidance and protective behaviors and the associated impacts based on focus group and literature review insights. All avoidance and protective behaviors and impacts reflected in the conceptual model were reported by ≥30% of participants (*n* = 14) or mentioned in all four focus group sessions conducted, with the exception of the two concepts introduced following the think tank session. ^a^ Highlighted as a key avoidance and protective behavior during the think tank result interpretation session. ^b^ Highlighted as a key negative impact during the think tank result interpretation session. ^c^ The think tank research partners noted that hoarding of products applied more at the beginning of the pandemic and is no longer relevant post-lockdown.

A conceptual model of salient avoidance and protective behaviors to avoid COVID-19 and the associated impacts on HRQoL and overall functioning is shown in Figure 3. This model consisted of 13 avoidance and protective behaviors to avoid COVID-19, categorized within four themes, and 13 impacts (11 negative and 2 positive) divided into five themes. Lack of trust in authorities/uncertainty over sources of information and hoarding of products and medications were listed as salient general societal impacts of the COVID-19 pandemic. Think tank research partners noted that hoarding applied more at the beginning of the pandemic and that supply chain issues were more relevant post-lockdown.

## 4. Discussion

To date, there has been a lack of qualitative data regarding the personal impact of specific COVID-19 avoidance and protective behaviors on individuals at high risk of severe COVID-19 in the post-lockdown period [32]. Findings from this study show that people at high risk of becoming severely ill from COVID-19 practice behaviors related to avoiding contact with others, such as staying at home, and avoiding physically going to shops, events, and gatherings. The use of masks, being vaccinated, being protected from COVID-19 by friends and family, relying on online delivery services, and having access to up-to-date information about COVID-19 infection rates were also mentioned. Participants often highlighted that avoidance behaviors negatively impacted family and social relationships, healthcare access, mental well-being, work, and finances. Some positive impacts included recognizing the importance of family and increased use of telehealth for medical care. The behaviors and impacts considered salient/important in the focus groups were also considered accurate by a think tank of three research partners. This culminated in the development of a conceptual model that will enhance understanding and facilitate discussion about avoidance and protective behaviors to avoid COVID-19. Hand washing and similar hygiene measures, as well as stigma and negative reactions of others to the behaviors still practiced by participants to avoid COVID-19, were not initially considered as salient/important during the focus groups but were added to the conceptual model based on findings from the think tank. 

In alignment with the findings from the current study, the sentiment among high-risk individuals, including participants of this study, was that they were protected during lockdown but, at the time of conducting this study, they felt left behind while the rest of the population have returned to ‘normal’ [6,19,20,21,22]. This is particularly important in the context of a recent systematic review on COVID-19 vaccines that demonstrated a lack of adequate vaccine protection among immunocompromised groups who remained at high risk of breakthrough infection and severe COVID-19 outcomes compared with the general population. Furthermore, these individuals were at greater risk of hospitalization and death following COVID-19 breakthrough infection [33]. Without any additional pharmacological interventions, people at high risk for severe COVID-19 will likely feel compelled to practice some level of physical distancing for many years to come, either upon the advice of their healthcare practitioner or of their own choosing. As such, it is important that health policy makers and researchers consider the unintended negative effects of physical distancing on HRQoL [3], mental well-being (psychological and emotional, due to increased levels of stress and anxiety) [6,9,34], work productivity [8], finances [35], and school performance (due to school absences) [36]. Previous studies have also highlighted negative impacts experienced by caregivers in their social and family relationships and emotional health due to social isolation [37]. In the current study, some participants reported needing to shut down their business due to having to isolate, which led to financial struggles, as well as the disruption of daily routines, social relationships, and emotional health. The disruption of financial markets and the economy due to the COVID-19 pandemic were also listed as negative impacts on income. 

It is noteworthy that the themes that emerged during these focus groups are very similar, both in terms of individual impacts and societal-level themes, to those noted in the All Party Parliamentary Group report, which investigated the psychological aspects of COVID-19 among immunocompromised people [6]. The concepts reported as salient by the participants in the focus groups were also in line with the recommendations by the US CDC and the UK Department for Health and Social Care [16,17], but despite the recommendations from these institutions, participants highlighted requiring more clarity over guidelines. Studies such as this one can be used to help define aspects that are of key importance for individuals at high risk of severe COVID-19, which in turn can be utilized in future guidance. 

Future research is warranted to see whether the findings from these focus groups can be replicated or furthered through qualitative and quantitative research to understand the extent to which COVID-19 remains a burden to high-risk individuals. The results from these focus groups will be used to inform the design of the observational EAGLE Study (AstraZeneca study number D8850R00013) [38,39].

### Strengths and Limitations of the Study

Individuals at high risk of severe COVID-19 and their caregivers were involved in each step of this exploratory study, including the interpretation of findings. This valuable input can help to inform future research, strategies, and recommendations. The validation of the study results by the think tank and the subsequent consolidation of study findings into the conceptual model, alongside the findings from the literature, provide a robust tool in a usable format to inform future research [40].

While the study sample size is reasonable given the qualitative nature of the study design, and included diverse geographic coverage, the size of the sample is nonetheless limited. This means that the avoidance and protective behaviors reported in this study should not be considered an exhaustive list, and similarly, the conceptual model may not reflect the most prominent behaviors or impacts of all high-risk individuals or their caregivers, as this was not developed based on concept saturation. However, given that this exploratory descriptive study was designed to guide future research, the numbers were considered appropriate to derive results capable of highlighting the continued burden hypothesized to be experienced by the high-risk and immunocompromised population in the post-lockdown COVID-19 period. Of note, the focus group sessions were limited to a one-hour duration and, as such, may have led to some concepts (particularly more spontaneous ideas) being unexplored and underrepresented. Moreover, we aimed to describe the pooled-group experience and did not aim to examine whether people with specific health conditions exhibited different behaviors. 

## 5. Conclusions

This exploratory study described the post-lockdown avoidance and protective behaviors undertaken by adults at high risk of severe COVID-19 and their caregivers. This study also captured the impacts of avoidance and protective behaviors, which, despite the lifting of government-imposed restrictions, are still practiced by many individuals with immunocompromising conditions and their caregivers. The conceptual model derived from this study will enable discussions with policy makers, payers, and health technology assessment bodies to facilitate access to strategies aimed at improving the quality of life of individuals at high risk of severe COVID-19, including immunocompromised individuals.

## Figures and Tables

**Table 1:** Summary of participant characteristics (*N* = 14).

Characteristic, *n* (%)	Patients (*n* = 12)	Caregivers (*n* = 2)
*Sex*		
Female	6 (50%)	2 (100%)
Male	6 (50%)	0
*Age (years)*		
18–30	0 (0%)	0 (0%)
31–50	3 (25%)	0 (0%)
51–64	5 (42%)	0 (0%)
65+	4 (33%)	0 (0%)
Missing	0 (0%)	2 (100%)
*Country of residence*		
United States	3 (25%)	2 (100%)
United Kingdom	6 (50%)	0
Canada	2 (17%)	0
Spain	1 (8%)	0
*Condition making the patient high-risk*		
Lupus	4 (33%)	0
Chronic kidney disease	3 (25%)	1 (50%) ^a^
Chronic obstructive pulmonary disease	2 (17%)	0
Liver cancer	1 (8%)	1 (50%) ^a^
Eosinophilic granulomatosis with polyangiitis	1 (8%)	0
Sickle cell ẞ-thalassemia	1 (8%)	0
*Relationship of caregiver*		
Wife	–	1 (50%)
Mother	–	1 (50%)

^a^ Indicates the condition of the person for whom the caregiver provides care.

## Data Availability

Data underlying the findings described in this manuscript may be obtained in accordance with AstraZeneca’s data sharing policy described at https://astrazenecagrouptrials.pharmacm.com/ST/Submission/Disclosure (accessed on 19 September 2024). Data for studies directly listed on Vivli can be requested through Vivli at www.vivli.org (accessed on 19 September 2024). Data for studies not listed on Vivli can be requested through Vivli at https://vivli.org/members/enquiries-about-studies-not-listed-on-the-vivli-platform/ (accessed on 19 September 2024). The AstraZeneca Vivli member page is also available, outlining further details: https://vivli.org/ourmember/astrazeneca/ (accessed on 19 September 2024).

## Appendix A

### Materials: Topics Explored in the Semi-Structured Focus Group Interview Guide

A full overview of the interview guide topics can be found below:

1. Meaning and description of avoidance and protective behaviors to avoid COVID-19
  - Types of avoidance and protective behaviors
  - Change of avoidance and protective behaviors over time
  - Recommendation to practice avoidance and protective behaviors
2. Impacts of avoidance and protective behaviors on participants’ health-related quality of life
  - Description of impacts of social isolation on emotional, social, family, work, school, and physical functioning
  - Negative and positive impacts experienced
  - Factors that impacted avoidance and protective behaviors
3. Factors influencing avoidance and protective behaviors
  - Internal and external factors impacting avoidance and protective behaviors
  - Facilitating and hindering factors for social isolation
  - Overarching impacts of the COVID-19 pandemic on society

**Table A1:** Domains and specific impacts of social isolation to avoid COVID-19 elicited from the literature review.

Domain	Impact Concepts
Social functioning	Collapse of social relationships Impact on religious traditions (e.g., inability to participate in religious celebrations) Increased dependency on technology
Emotional functioning	Frustration Loneliness/isolation Anxiety/distress/worry Loss of motivation Concern Stress Fear and uncertainty Depression/sadness Anger/bad temper Sense of shock, chaos, and confusion Loss and grief Stigma Guilt Loss of track of time Burnout and parental stress
Work and financial impacts	Precarious employment and job instability Productivity loss at work/school Redefinition of roles within occupations Obligation to work home-based Disruption to college/academic education Unmet education outcomes Uncertainty over educational plans
Family functioning	Impact on family relations and interactions Impact on living arrangements and inadequate housing conditions Impact on interpersonal relationships Loss of social contact with family members due to their vulnerability Disrupted routines
Physical functioning	Increase in sedentary behaviors/lower levels of physical activity Inability to do physical hobbies Impact on sleep/insomnia

**Table A2:** Overall impacts of the COVID-19 pandemic on society elicited from the literature review.

Domain	Impact Concepts
Social functioning	Lack of trust in institutions (e.g., government bodies, scientific institutions, pharmaceutical companies) Lack of trust in guidelines Lack of trust in media/communication Lack of trust in other people’s judgment Increase in substance abuse Increase in gender-based violence Increased perception of inequality Limited movement/freedom Hoarding of products and medicines Worsening of eating habits Increased generation of waste
Healthcare access impacts	Overfocus on COVID-19 in hospitals Difficulties accessing healthcare services

**Table A3:** Frequency of avoidance and protective behaviors mentioned by participants and across the four focus group sessions.

Avoidance and Protective Behaviors, *n* (%)	Total Participant Sample (*N* = 14)	Focus Group Sessions *N* = 4)
Wearing a mask	11 (79)	4 (100)
Home delivery of essentials (food, groceries)	9 (64)	4 (100)
Staying home	9 (64)	3 (75)
Avoiding bystanders/crowds	8 (57)	4 (100)
Family members’ support and protective behaviors	8 (57)	4 (100)
Being vaccinated	8 (57)	3 (75)
Avoiding going to parties/events/family gatherings	6 (43)	3 (75)
Avoiding going to shopping facilities	6 (43)	4 (100)
Reliance on data/statistics to make decisions	5 (36)	4 (100)
Restricting visits to the household	5 (36)	2 (50)
Meeting others through video apps	4 (29)	4 (100)
Meeting others outdoors	4 (29)	4 (100)
Avoiding in-person contacts/meetings	4 (29)	3 (75)
Isolation within the household	3 (21)	1 (25)
Taking precautions when near people	3 (21)	2 (50)
Government-issued delivery of goods	3 (21)	3 (75)
Requiring others who live with you to be careful/isolate	3 (21)	3 (75)
Washing hands	3 (21)	3 (75)
Avoiding sick people	3 (21)	2 (50)
Avoiding going to restaurants	2 (14)	1 (25)
Taking precautions when in contact with exposed people	2 (14)	2 (50)
Disinfection of all products (e.g., groceries)	2 (14)	2 (50)
Going to small shops	2 (14)	2 (50)
Going to big shopping facilities when not busy	2 (14)	2 (50)
Requesting others to take tests before visits	2 (14)	2 (50)
Choosing to miss healthcare appointments	1 (7)	1 (25)
Avoiding hotels when traveling	1 (7)	1 (25)
Avoiding traveling	1 (7)	1 (25)
Advanced planning for going out	1 (7)	1 (25)
Contacting others to increase passive immunity	1 (7)	1 (25)
Avoiding touching objects	1 (7)	1 (25)
Taking immune boosters and vitamins (e.g., vitamin C, ginger tea)	1 (7)	1 (25)
Holding one’s breath when close to others	1 (7)	1 (25)

Concepts highlighted in grey indicate salient concepts reported by ≥30% of participants or mentioned by at least one patient or caregiver in all separate four focus group sessions.

**Table A4:** Frequency of all avoidance and protective behaviors mentioned by caregivers.

Avoidance and Protective Behaviors, *n* (%)	Caregivers (*N* = 2)
Avoiding bystanders/crowds	2 (100)
Family members’ support and protective behaviors	2 (100)
Meeting others outdoors	2 (100)
Wearing a mask	2 (100)
Home delivery of essentials (food, groceries)	2 (100)
Avoiding going to restaurants	1 (50)
Avoiding hotels when traveling	1 (50)
Avoiding going to parties/events/family gatherings	1 (50)
Taking precautions when near people	1 (50)
Avoiding in-person contacts/meetings	1 (50)
Meeting others through video apps	1 (50)
Being vaccinated	1 (50)
Avoiding sick people	1 (50)
Restricting who comes into the home	1 (50)
Staying home	1 (50)

Concepts highlighted in grey indicate concepts reported by both caregivers.

Participants were asked to consider the impacts of their avoidance and protective behaviors to avoid COVID-19. Impacts were defined as how the avoidance and protective behaviors affected their overall HRQoL, including their family, social, emotional, physical and work- or school-related functioning. Participants highlighted negative and positive impacts of their avoidance and protective behaviors to avoid COVID-19 on their HRQoL.

**Table A5:** Frequencies of all impacts of avoidance and protective behaviors to avoid COVID-19 on all participants’ HRQoL.

Impact, *n* (%)	Total Participant Sample (*N* = 14)	Focus Group Sessions (*N* = 4)
Negative impacts		
Family functioning		
Problems with family relationships (e.g., family conflicts) or missing family interactions (e.g., weddings, funerals)	10 (71)	4 (100)
Living arrangements/lack of adequate housing	3 (21)	1 (25)
Disrupted routines	2 (14)	2 (50)
Loss of contact with vulnerable family members	1 (7)	1 (25)
Partner relationships	1 (7)	1 (25)
Social functioning		
Impact on social relations/collapse of social contacts	8 (57)	4 (100)
Difficulties accessing shopping services	3 (21)	2 (50)
Overreliance on others	1 (7)	1 (25)
Emotional functioning		
Anxiety/distress/worry	7 (50)	3 (75)
Fear and uncertainty	6 (43)	3 (75)
Depression/sadness	5 (36)	3 (75)
Loneliness/isolation	5 (36)	3 (75)
Fatigue due to reliance on virtual meetings for social contact	4 (29)	2 (50)
Frustration	4 (29)	2 (50)
Loss of confidence in own abilities	3 (21)	1 (25)
Feeling of being forgotten	3 (21)	3 (75)
Sense of shock and chaos/confusion	3 (21)	2 (50)
Emotional well-being problems (unspecified)	3 (21)	3 (75)
Anger/bad temper	2 (14)	1 (25)
Stress	2 (14)	2 (50)
Burnout and parental stress	1 (7)	1 (25)
Guilt (due to transmitting COVID; not being able to help)	1 (7)	1 (25)
Loss and grief	1 (7)	1 (25)
Concern	1 (7)	1 (25)
Loss of motivation	1 (7)	1 (25)
Work- and financial-related		
Precarious employment and job instability	5 (36)	4 (100)
Loss of income and financial problems	5 (36)	4 (100)
Disruption to college/school life	4 (29)	2 (50)
Having to work home-based	3 (21)	3 (75)
Difficulty establishing relationships with peers/professors	3 (21)	2 (50)
Productivity loss in studying	2 (14)	2 (50)
Redefinition of roles within occupations	1 (7)	1 (25)
Unmet economic needs	1 (7)	1 (25)
Moving to home-based classes	1 (7)	1 (25)
Unmet education goals	1 (7)	1 (25)
Healthcare access		
Difficulty accessing healthcare/lack of proper healthcare support	6 (43)	3 (75)
Isolation during doctor visits	5 (36)	2 (50)
Physical functioning		
Lower levels of physical activity	4 (29)	3 (75)
Walking problems due to home isolation/lack of activity	2 (14)	2 (50)
Inability to do (physical, outdoor) hobbies	1 (7)	1 (25)
Positive impacts		
Family functioning		
Recognition of the importance of family	5 (36)	4 (100)
Healthcare access		
Increased use of telehealth options	8 (57)	4 (100)
More engaged in own health/healthcare	2 (14)	2 (50)
Social functioning		
Initiating new activities/learning new things	3 (21)	2 (50)
Ability to meet new people virtually	1 (7)	1 (25)
Work- and financial-related		
Increase in productivity due to telework	2 (14)	1 (25)
Increased opportunities to do charity/voluntary work	2 (14)	2 (50)
Increased flexibility in work schedules	1 (7)	1 (25)
Development of new remote learning strategies	1 (7)	1 (25)
Physical functioning		
Increased focus on physical health	1 (7)	1 (25)

Concepts highlighted in grey indicate salient concepts reported by ≥30% of participants or mentioned by at least one patient or caregiver in all separate four focus group sessions.

A total of 25 impacts of avoidance and protective behaviors to avoid COVID-19 and overall impacts of COVID-19 on society were reported by the two caregivers interviewed during the focus groups. A total of five impacts were reported by both caregivers, one of which was a family functioning impact (family relationships and interaction problems), three of which were emotional functioning impacts (depression and sadness; stress; and anxiety, distress, and worry), and one of which was a negative overall impact of the COVID-19 pandemic on society (loss of loved ones).

**Table A6:** Frequencies of all impacts of avoidance and protective behaviors to avoid COVID-19 on caregiver’s HRQoL.

Impact, *n* (%)	Caregivers (*N* = 2)
Negative impacts	
Family functioning	
Problems with family relationships (e.g., family conflicts) or missing family interactions (e.g., weddings, funerals)	2 (100)
Social functioning	
Impact on social relations/collapse of social contacts	1 (50)
Emotional functioning	
Depression/sadness	2 (100)
Stress	2 (100)
Anxiety/distress/worry	2 (100)
Feeling of being forgotten	1 (50)
Burnout and parental stress	1 (50)
Sense of shock and chaos/confusion	1 (50)
Fear and uncertainty	1 (50)
Loneliness/isolation	1 (50)
Work- and financial-related	
Precarious employment and job instability	1 (50)
Loss of income and financial problems	1 (50)
Healthcare access impacts	
Difficulty accessing healthcare/lack of proper support	1 (50)
Isolation during doctor visits	1 (50)
Physical impacts	
Walking problems due to home isolation/lack of activity	1 (50)
Inability to do (physical, outdoor) hobbies	1 (50)
Lower levels of physical activity	1 (50)
Positive impacts	
Family functioning	
Recognition of the importance of family	1 (50)
Healthcare access impacts	
More engaged in own health/healthcare	1 (50)
Increased use of telehealth options	1 (50)

Concepts highlighted in grey indicate concepts reported by both caregivers.
